# Data-Driven Modeling of Src Control on the Mitochondrial Pathway of Apoptosis: Implication for Anticancer Therapy Optimization

**DOI:** 10.1371/journal.pcbi.1003011

**Published:** 2013-04-04

**Authors:** Annabelle Ballesta, Jonathan Lopez, Nikolay Popgeorgiev, Philippe Gonzalo, Marie Doumic, Germain Gillet

**Affiliations:** 1 BANG project team, INRIA Rocquencourt, Le Chesnay, Yvelines, France; 2 Centre de Recherche en Cancérologie de Lyon, INSERM U1052/CNRS UMR5286, Lyon, Rhône, France; 3 Université de Lyon - Université Claude Bernard Lyon 1, Lyon, Rhône, France; 4 Hospices Civils de Lyon - Fédération de Biochimie Nord - Hôpital de la Croix Rousse, Lyon, Rhône, France; Northeastern University, United States of America

## Abstract

Src tyrosine kinases are deregulated in numerous cancers and may favor tumorigenesis and tumor progression. We previously described that Src activation in NIH-3T3 mouse fibroblasts promoted cell resistance to apoptosis. Indeed, Src was found to accelerate the degradation of the pro-apoptotic BH3-only protein Bik and compromised Bax activation as well as subsequent mitochondrial outer membrane permeabilization. The present study undertook a systems biomedicine approach to design optimal anticancer therapeutic strategies using Src-transformed and parental fibroblasts as a biological model. First, a mathematical model of Bik kinetics was designed and fitted to biological data. It guided further experimental investigation that showed that Bik total amount remained constant during staurosporine exposure, and suggested that Bik protein might undergo activation to induce apoptosis. Then, a mathematical model of the mitochondrial pathway of apoptosis was designed and fitted to experimental results. It showed that Src inhibitors could circumvent resistance to apoptosis in Src-transformed cells but gave no specific advantage to parental cells. In addition, it predicted that inhibitors of Bcl-2 antiapoptotic proteins such as ABT-737 should not be used in this biological system in which apoptosis resistance relied on the deficiency of an apoptosis accelerator but not on the overexpression of an apoptosis inhibitor, which was experimentally verified. Finally, we designed theoretically optimal therapeutic strategies using the data-calibrated model. All of them relied on the observed Bax overexpression in Src-transformed cells compared to parental fibroblasts. Indeed, they all involved Bax downregulation such that Bax levels would still be high enough to induce apoptosis in Src-transformed cells but not in parental ones. Efficacy of this counterintuitive therapeutic strategy was further experimentally validated. Thus, the use of Bax inhibitors might be an unexpected way to specifically target cancer cells with deregulated Src tyrosine kinase activity.

## Author Summary

Personalizing medicine on a molecular basis has proven its clinical benefits. The molecular study of the patient's tumor and healthy tissues allowed the identification of determinant mutations and the subsequent optimization of healthy and cancer cells specific response to treatments. Here, we propose a combined mathematical and experimental approach for the design of optimal therapeutics strategies tailored to the patient molecular profile. As an *in vitro* proof of concept, we used parental and Src-transformed NIH-3T3 fibroblasts as a biological model. Experimental study at a molecular level of those two cell populations demonstrated differences in the gene expression of key-controllers of the mitochondrial pathway of apoptosis thus suggesting potential therapeutic targets. Molecular mathematical models were built and fitted to existing experimental data. They guided further experimental investigation of the kinetics of the mitochondrial pathway of apoptosis which allowed their refinement. Finally, optimization procedures were applied to those data-calibrated models to determine theoretically optimal therapeutic strategies that would maximize the anticancer efficacy on Src-transformed cells under the constraint of a maximal allowed toxicity on parental cells.

## Introduction

Protein tyrosine kinases of the Src family are involved in multiple facets of cell physiology including survival, proliferation, motility and adhesion [1]. Their deregulation has been described in numerous malignancies such as colorectal, breast, melanoma, prostate, lung or pancreatic cancers and is known to favor tumorigenesis and tumor progression [2]–[4].

Modulation of apoptosis sensitivity by Src deregulation is more controversial. We recently described that Src activation promotes resistance to the mitochondrial pathway of apoptosis in mouse and human cancer cell lines [5]. The molecular mechanism underlying such resistance involved the accelerated degradation of the proapoptotic BH3-only protein Bik. Indeed, in Src-transformed NIH 3T3 mouse fibroblasts, Bik was found to be phosphorylated by activated Erk1/2, which was followed by Bik subsequent polyubiquitylation and proteasomal degradation [5]. Thus in Src-transformed cells, Bik downregulation compromised Bax activation and mitochondrial outer membrane (MOM) permeabilization upon an apoptotic stress [5].

That observation might be of importance since MOM permeabilization is the key step that commits cells to apoptosis. Indeed, MOM permeabilization leads to the irreversible release of cytochrome c and other cytotoxic molecules from the mitochondrial inter-membrane space into the cytosol [6], [7]. Once released, cytochrome c induces the formation of the apoptosome complex, which triggers caspase activation, these molecules being the main executioners of the apoptotic program. MOM permeabilization is triggered by the insertion and oligomerization of the pro-apoptotic effector Bax into the membrane [8]–[11]. Antiapoptotic proteins such as Bcl-2 or Bcl-xL prevent this process, whereas pro-apoptotic BH3-only proteins contribute to Bax activation [6], [11]–[16]. Using western blotting and specific shRNAs, the respective contribution of the different Bcl-2 family members to the cell response triggered by various death- inducing agents was assessed in parental and Src-transformed NIH-3T3 fibroblasts [5].

Experimentally and mathematically investigating the cell response to death-inducing agents might be of interest since it has long been postulated that restoration of apoptosis might be an effective way to selectively kill cancer cells. The rationale of this assumption is that cancer cells need to counteract the pro-apoptotic effect of oncogenes such as Myc or E2F-1 that stimulate cell proliferation as well [17]. Moreover, Src deregulation has specifically been associated with resistance to treatment in a number of cancers [18], [19]. Therefore, a critical clinical concern lies in the design of therapeutic strategies that would circumvent resistance to apoptosis of cells with deregulated Src activity. To this end, several classes of therapeutic agents might be *a priori* considered. Inhibitors of Src tyrosine kinases, such as dasatinib, are currently widely used in the clinic [20]–[23]. Other anticancer therapeutic strategies aim at restoring apoptosis in cancer cells [24]. In particular, inhibitors of antiapoptotic proteins such as ABT-737 or the Oblimersen Bcl-2 antisense oligodeoxyribonucleotide are currently evaluated in clinical trials [25]–[28]. Apoptosis may also be restored by increasing the expression of pro-apoptotic proteins such as Bax, Bik or p53 [29]–[32].

Here we propose a systems biology approach for optimizing potential anticancer therapeutic strategies using parental and Src-transformed NIH 3T3 fibroblasts as a biological model. To this end, molecular mathematical models of Bik kinetics and of the mitochondrial pathway of apoptosis were built and fitted to available experimental data. They guided further experimental investigation in parental and Src-transformed cells which allowed their refinement. Then, those models were used to generate predictions which were validated by subsequent specifically-designed experiments. Finally, we theoretically explored different drug combinations involving the kinase inhibitor staurosporine, Src inhibitors, and activators or inhibitors of the Bcl-2 protein family, in order to design optimal anticancer strategies for this biological system. Optimal strategies were defined as those which maximized the efficacy on Src-transformed cells considered as cancer cells under the constraint of toxicity remaining under a tolerable threshold in parental cells.

## Results

### Bik kinetics in non-apoptotic conditions

We recently provided evidence that Bik, a BH3-only protein, is a key regulator of apoptosis in the considered biological system [5]. Therefore we first built a mathematical model to investigate Bik kinetics in non-apoptotic conditions. Bik concentration temporal variations were assumed to result from two processes: protein formation and protein polyubiquitylation, which eventually leads to its degradation. Let us denote 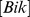 and 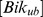 the intracellular concentration of Bik and polyubiquitylated Bik proteins respectively, expressed in nM.

**Figure 1:**
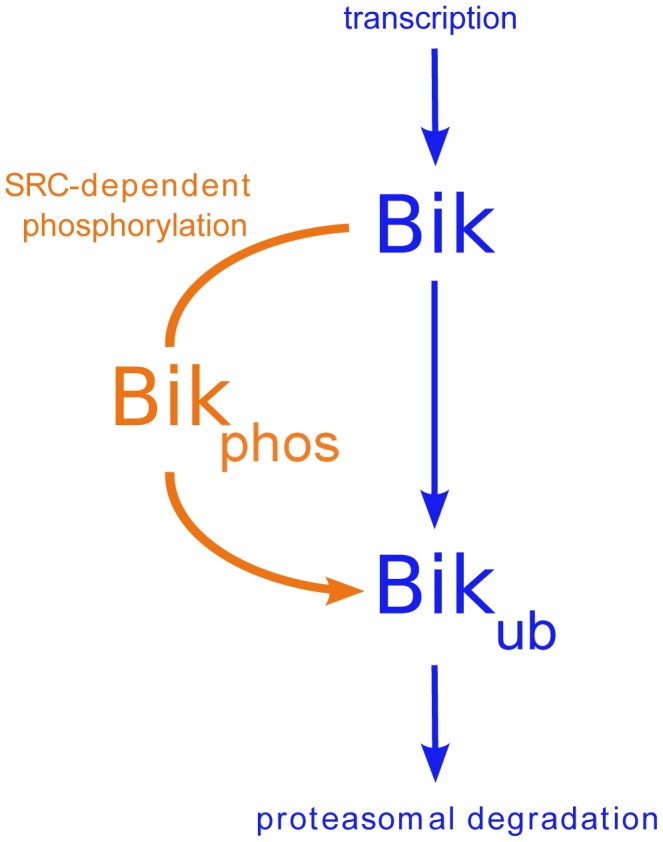
Bik kinetics in non-apoptotic conditions. 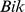
 proteins are polyubiquitylated (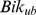) prior to their proteasomal degradation. This ubiquitylation occurs spontaneously in parental NIH-3T3 cells and is enhanced in Src-transformed cells through Src-dependent activation of Erk1/2 kinases which accelerate Bik phosphorylation (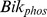), thus favoring its ubiquitylation.

Bik protein was assumed to be synthesized at a constant rate 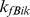 in both Src-transformed and parental cells as suggested by similar Bik mRNA level in both cell types [5]. Concerning Bik ubiquitylation, we considered that it occurred either spontaneously at the rate 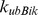, or after Bik phosphorylation by activated Erk1/2 downstream of SRC activation, as demonstrated in Src-transformed fibroblasts ([5], Figure 1). In those cells, this prior phosphorylation increased Bik ubiquitylation rate and further proteasomal degradation. This Src-dependent pathway was modeled by Michaelis-Menten kinetics with parameters 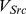 and 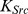. In the model, we assumed that spontaneous and Src-mediated ubiquitylation could occur in both transformed and parental cells. Ubiquitin molecules were assumed to be in large excess compared to Bik amount. Therefore ubiquitin concentration was considered as constant and implicitly included in 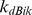 and 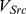.

Poly-ubiquitylated molecules 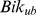 were then assumed to be degraded by the proteasome at a constant rate 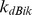 in both cell types. 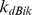 was arbitrary set to 1 as it does not influence 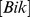 kinetics, and only acts on 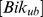. The model of Bik kinetics can be written as follows:
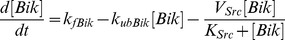
(1)

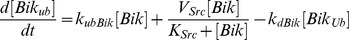
(2)


**Figure 2:**
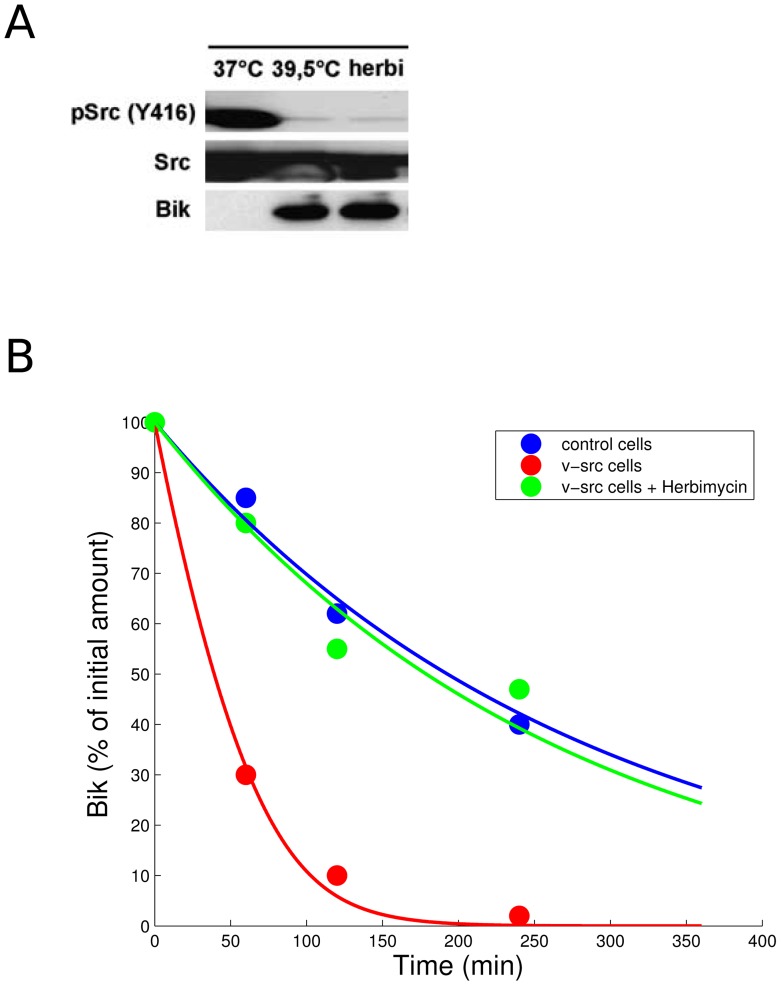
Estimating Bik degradation rates in control and Src-transformed cells. **A** Endogenous Bik level was compared with Src level and Src kinase activity as monitored by Y416 phosphorylation in Src-transformed cells. v-Src kinase activity was inhibited either by herbimycin or by incubation at 39.5°C. Src inhibition by herbimycin was quantified to 98%. Reprinted with permission from [5] **B** Bik degradation was monitored in parental and Src-transformed cells. v-Src kinase activity was inhibited by herbimycin in transformed cells. Dots are experimental results reprinted from [5]. Solid lines represent the best fit of Bik kinetics mathematical model (Equations 1 and 2). See Results for parameter values.

Parameters were then estimated for parental and Src-transformed cells by fitting experimental results on Bik protein degradation in both cell types ([5], reprinted with permission in Figure 2B). We assumed that the spontaneous phosphorylation occurred at the same rate in parental and Src-transformed fibroblasts and therefore looked for a unique 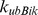. Parameters of Src-dependent Bik degradation were denoted 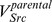 and 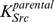 in parental cells and 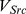 and 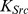 in Src-transformed 3T3 cells. Inhibition of Src kinase activity by herbimycin was experimentally monitored in Src-transformed cells (Figure 2A, reprinted with permission from [5]). Herbimycin exposure achieved a decrease of 98% in phosphorylated Y416 amount. Therefore, we modeled herbimycin exposure as a decrease of 98% in 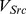 values. See Text S1 for details on the parameter estimation procedure.

The best-fit parameter value for the spontaneous ubiquitylation was 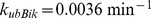. Src-dependent ubiquitylation was predicted to be inactive in normal fibroblasts as 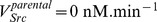, which was in agreement with experimental results [5]. On the contrary, the Src pathway was predominant in transformed cells as 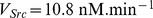 and 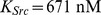 which leads to 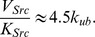


The dynamical system 1–2 admits a unique steady state:
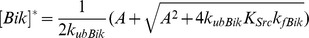


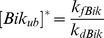
where 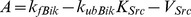. For parental cells, in which 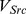 is equal to zero, 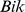 steady state becomes 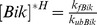.

**Figure 3:**
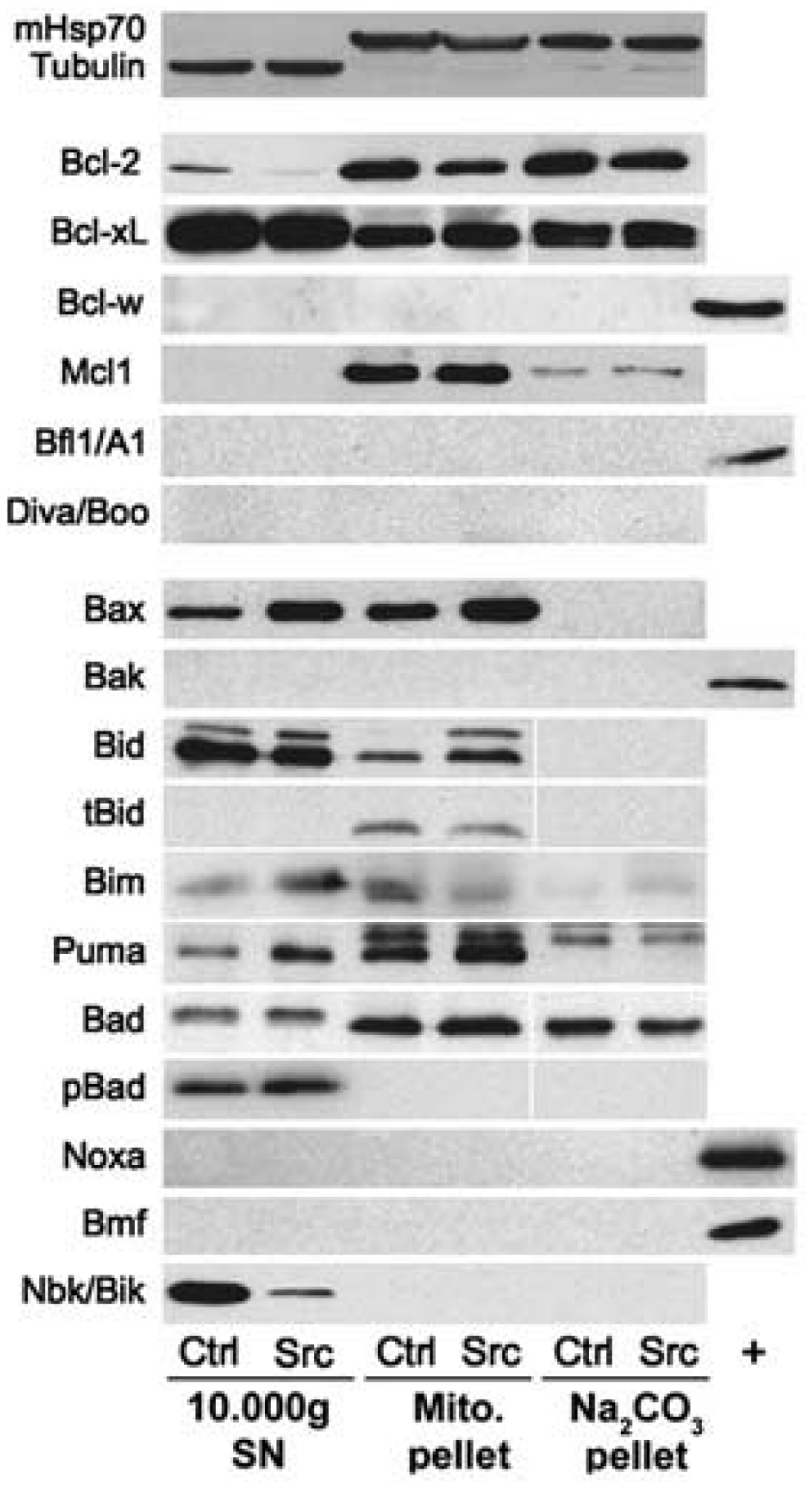
Western blotting analyses of the Bcl-2 family proteins in control (Ctrl) and v-src-transformed cells (Src). Differential centrifugation was used for cell fractionation. Each experiment was repeated at least twice. Mitochondrial Hsp70 and a-tubulin were used as loading controls for mitochondrial fractions and 10 000× g supernatants (10 000 g SN), respectively (loading 100 mg proteins). Whole cell lysates from HeLa (Bcl-w), BP3 (Bfl1/A1), 293T (Noxa and Bmf) and WT MEF (Bak) cells were used as positive controls when proteins were undetected. Data were reprinted with permission from [5].

Bik steady-state concentrations in parental cells was assumed to be equal to 50 nM which is in the physiological range of BH3-only protein intracellular levels [33]–[37]. This allowed us to deduce 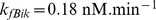. We then computed Bik steady-state concentrations in Src-transformed cells which was equal to 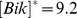 nM. Thus, the simulated ratio of Bik concentration in Src-transformed cells over that in parental cells was equal to 0.18 which is similar to the experimentally-observed value quantified to 0.2 (Figure 3, Table 1). This constitutes a partial validation of the model since the data of Figure 3 was not used in Bik kinetics model design and calibration. In the following, these steady state concentrations were used as Bik initial condition since cells were assumed to be in non-apoptotic conditions prior to the death stimulus.

**Table 1:** Ratios of the different BCL-2 family proteins involved in staurosporine-induced apoptosis.

	Parental cells (nM)	Src-transformed cells (nM)	Experimentally-determined ratio Src-transformed/parental	Simulated ratio Src-transformed/parental
Bik	50	9.2	0.2	0.18
Bax	48	100	2.1	-
Bcl-2+ Bcl-xL +Mcl-1	545	600	1.1	-
Bid	52	40	0.77	-
tBid	1	1	1	-

These values were calculated from the experimental measurements of the Bcl-2 family proteins (Figure 3). See Results section for details.

### Bik kinetics upon apoptotic stress

We then investigated Bik kinetics in parental and Src-transformed NIH-3T3 cells in response to an apoptotic stress that consisted of a 8-hour-long exposure to staurosporine (2 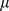
M). As demonstrated by knockdown experiments (Figure 2b in [5]), Bik was required for apoptosis induction. Bik was present in non-transformed cells with no sign of apoptosis in normal conditions, which suggested either that Bik concentration was not large enough to trigger apoptosis in these conditions, or that Bik was activated upon apoptotic stress.

**Figure 4:**
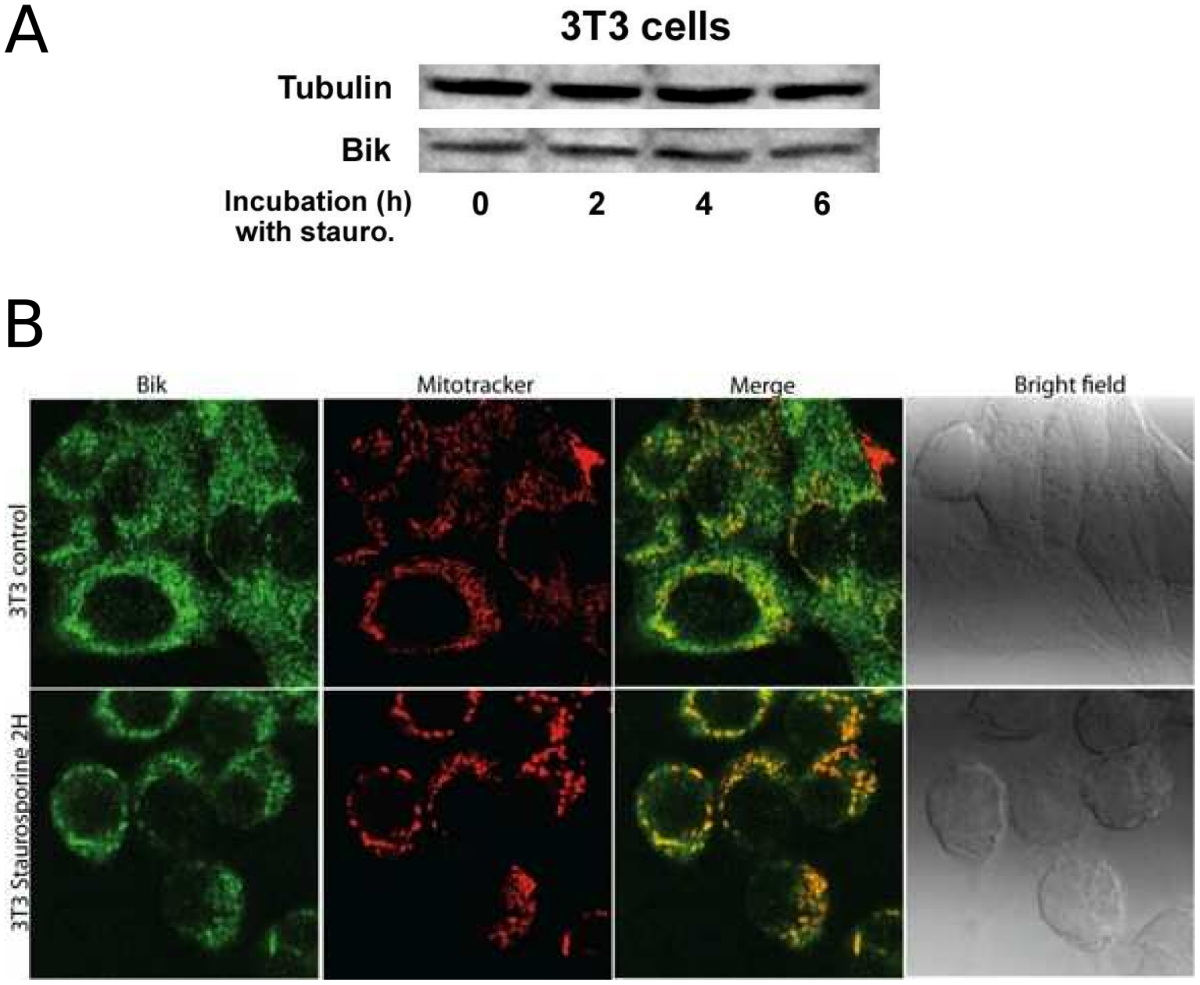
Bik behavior after a death stimulus. **A** Bik immunoblotting in presence of staurosporine in parental NIH-3T3 cells. Bik protein amount remained constant during apoptosis. **B** Bik relocalized from ER to mitochondria during staurosporine exposure in parental NIH-3T3 cells. Mitochondria were labeled by Mitotracker (red). Endogenous Bik was tracked by an anti-Bik-BH3 and a secondary antibody labeled with FITC (green). Yellow indicated colocalization.

The first assumption to be mathematically investigated was that Bik protein amount might increase upon staurosporine treatment as a result of the turning-off of the degradation processes, Bik synthesis rate remaining unchanged under staurosporine treatment. Thus, if Bik ubiquitylation process is turned off in the model, only the formation term remains in equation 1 which is now the same for parental and transformed cells, with different initial conditions. This equation can be solved analytically:
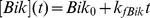
(3)where 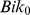 stands for Bik initial concentration taken equal to Bik steady state concentration in parental and transformed fibroblasts. We did not observe any significant apoptosis either in normal or Src-transformed cells in the first six hours of staurosporine treatment (data not shown). In non-transformed cells, setting t = 360 min in equation 3 gave 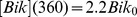 which meant that Bik concentration would only double in six hours if this hypothesis was right. This was tested by measuring Bik protein level during staurosporine exposure in parental cells. However, no significant increase in Bik levels upon a 6 hour-long staurosporine treatment was observed, which ruled out that the induction of apoptosis could depend on Bik accumulation (Figure 4 A).

Therefore, we investigated a second hypothesis that consisted of an activation of Bik upon apoptosis induction. Such a possibility might rely on a release of Bik from a protein complex upon apoptotic stress as observed with other BH3-only proteins such as Bad, Bim or Bmf [38]. To investigate the likelihood of this hypothesis, we performed the immunostaining of endogenous Bik in parental NIH-3T3 cells upon staurosporine exposure. Our data was in agreement with a relocation of Bik from its known location at the ER to the mitochondria within 2 h of treatment ([39], [40], Figure 4 B). This relocation might correspond to Bik release from a binding protein at the ER as previously observed [41]. We modeled this relocation by the equations 4 and 5 in which 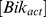 stands for Bik protein that had been activated possibly through this relocation and 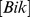 represents inactive Bik molecules. This translocation occurred at the rate 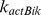. Colocalization between Bik fluorescence and mitotracker staining showed that 45
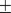
13% of Bik molecules were located at the mitochondria within 2 h of treatment which led to the estimated value 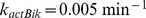 (Figure 4 B).

### Designing a mathematical model of the mitochondrial pathway of apoptosis in NIH-3T3 fibroblasts

We then investigated the mitochondrial pathway of apoptosis in NIH-3T3 parental and Src-transformed cells. We only considered the Bcl-2 members that were experimentally detected in this biological model [5]. The only pro-apoptotic multidomain effector was Bax, whereas the multidomain antiapoptotic protein family was represented by Bcl-2, Bcl-xL and Mcl-1 [5]. Five BH3-only proteins were present: three BH3-only activators (i.e. able to directly bind and activate Bax) Puma, Bim and tBid and two BH3-only sensitizers (i.e. able to bind Bcl-2 and related apoptosis inhibitors, but unable to bind and activate Bax) Bad and Bik. The respective role of present BH3-only proteins in apoptosis induction was assessed by a shRNA-mediated approach.

Bim, which was expressed at very low level, could be neglected in the onset of apoptosis, since its downregulation induced no significant increase in apoptosis resistance upon staurosporine, thapsigargin or etoposide. In contrast, PUMA had a prominent role for apoptosis induced by genotoxic stresses (UV or etoposide) but displayed no significant role in staurosporine- and thapsigargin-induced apoptosis [5]. As we focused here on staurosporine-induced apoptosis, the only BH3-only activator that we considered was tBid. Concerning BH3-only sensitizers, Bad could be neglected as its silencing by shRNA did not significantly modify cell response to staurosporine. Therefore the only sensitizer to be considered was Bik.

Bax, Bik and tBid were described to bind all the antiapoptotic proteins expressed in our biological model, namely Bcl2, Bcl-xL and Mcl-1. Thus, for the sake of simplicity, we denoted by 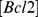 the cumulative concentration of those three antiapoptotic proteins.

We then modeled Bax activation. In non-apoptotic conditions, Bax spontaneously adopts a closed 3D-conformation that does not bind antiapoptotic proteins [10]. This conformation was denoted 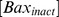. During apoptosis, Bax transforms into an opened 3D-conformation (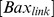) and inserts strongly into the MOM. 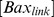 molecules can be inhibited by 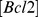 antiapoptotic proteins which trap them into 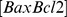 dimers. Moreover, they may spontaneously transform back into their closed conformation 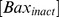 [42].

If they are not inhibited, 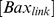 molecules may oligomerize into 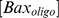 molecules and create pores in the MOM which correlates with the release into the cytosol of apoptogenic factors, including cytochrome C [8]–[10]. We considered that 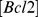 was inefficient at binding oligomerized Bax [7]. In the model, Bax oligomerization happens either by the oligomerization of two 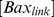 molecules or by a much faster autocatalytic process in which a 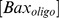 molecule recruits a 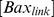 molecule to create two 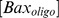 molecules. Those two processes occurred at the respective rates 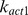 and 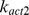 which were chosen such that 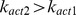 to account for the preponderance of the autocatalytic pathway.

Bax activation from 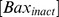 into 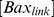 isoforms was assumed to be catalyzed by the BH3-only activator 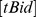. We assumed that this reaction occurred in a “kiss and run” manner and therefore follows Michaelis-Menten kinetics. 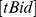 resulted from 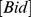 activation by truncation which occurred at the rate 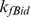 [43]. BH3-only activator 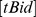 can also be inhibited by 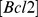 which trap it into 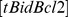 complexes. Those complexes may be dissociated by active Bik molecules 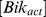 which bind to 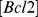 and release 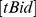 [13]. Finally, we also considered that 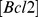 antiapoptotic proteins directly inhibit active Bik molecules and associate into 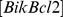 complexes.

**Figure 5:**
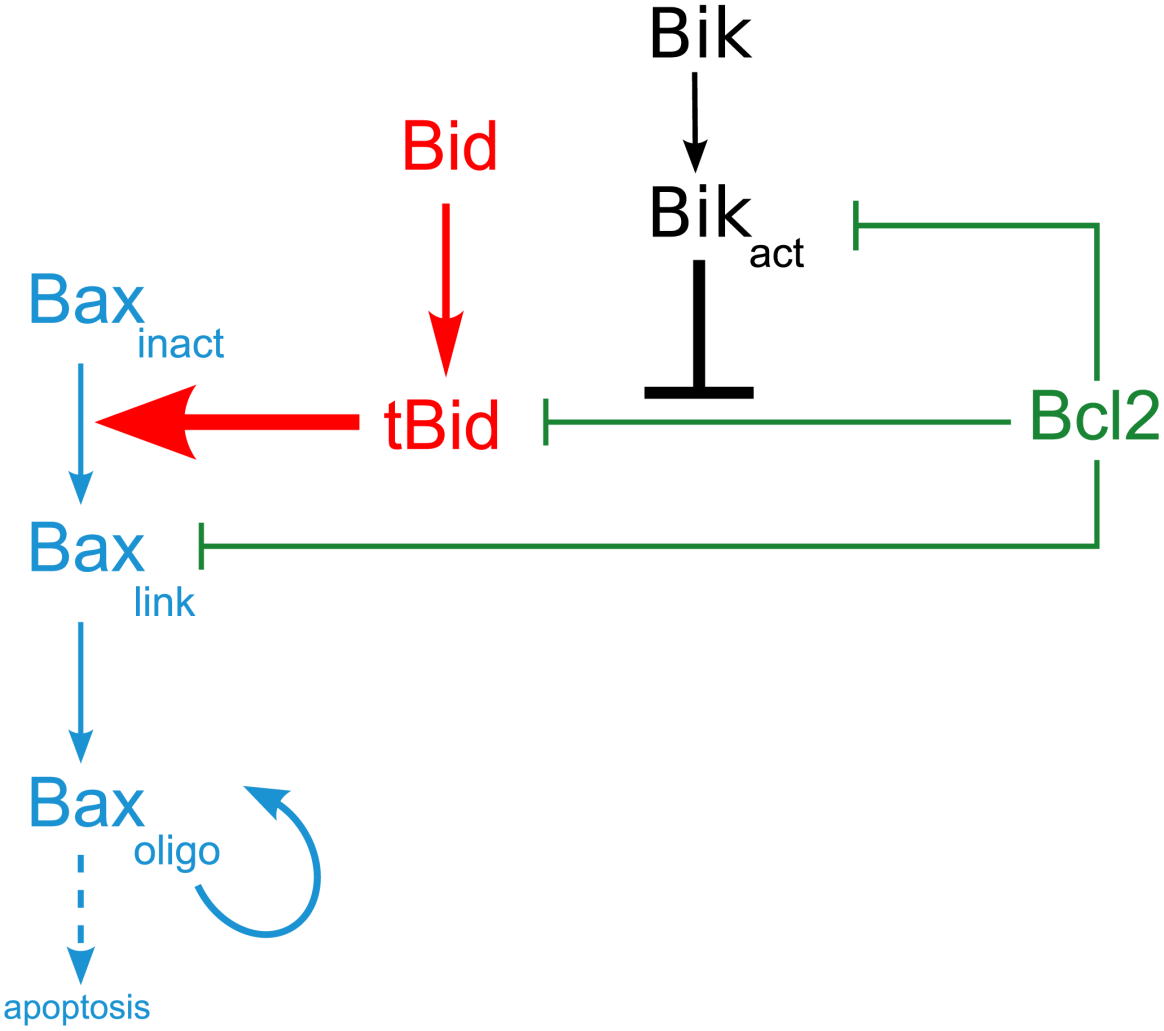
Considered molecular pathways of mitochondrial apoptosis. 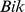
 is activated upon death stimulus (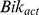) and inhibits Bcl2 antiapoptotic activity on BH3 activator 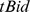. Free 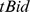 then activates closed Bax molecules 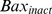 into 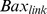 which binds to the mitochondrial membrane. 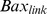 molecules then oligomerize with each other (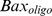) in an autocatalytic process. Oligomerized Bax molecules create pores in the mitochondrial membrane which allow the release of apoptogenic factors in the cytosol, leading the cell to apoptosis. Bcl2 also inhibits 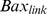 and 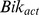.

Above-mentioned chemical reactions that occur spontaneously were assumed to follow the law of mass action. All protein concentrations are expressed in nM in the mathematical model. This mathematical model is recapitulated in Figure 5 and Table 2. It can be written as follows:
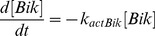
(4)

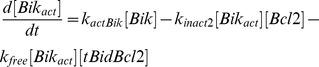
(5)

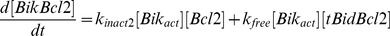
(6)

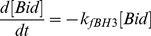
(7)

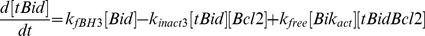
(8)

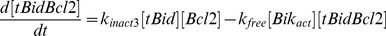
(9)

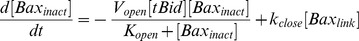
(10)

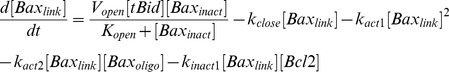
(11)

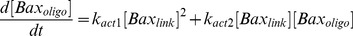
(12)

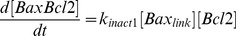
(13)

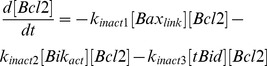
(14)


**Table 2:** Parameters values for the apoptosis model.

No.	Reaction	Definition	Parameter	Value
1	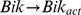	Bik migration from ER to mitochondria	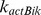 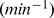	0.005
2	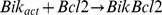	Inhibition of Bik by Bcl2	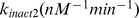	0.000105
3	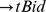	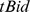 formation during apoptosis	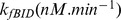	0.00014
4	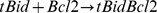	Inhibition of 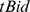 by Bcl2	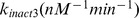	0.000087
5	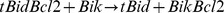	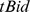 release from complexes with Bcl2 by Bik	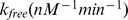	0.88
6	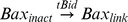	Bax activation by 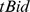	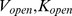 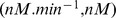	0.0104, 114
7	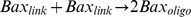	Oligomerization of two Bax monomers	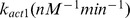	0.000185
8	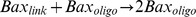	Bax autocatalytic oligomerization	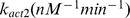	0.0144
9	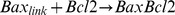	Inhibition of Bax by Bcl2	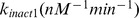	0.000021
10	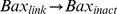	Bax spontaneous inactivation	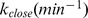	0.007
11	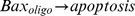	Apoptosis triggering by 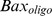	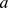 , 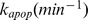	4.15, 0.0205

Parameters were fitted to experimental data as described in the Results section and in Text S1.

Bik total protein amount was assumed to be constant during apoptosis as experimentally demonstrated (Figure 4 A). We also assumed that 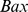, 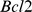 and 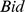 total amounts remained constant following the death stimulus. However, the apoptotic stress may induce Bax transcription and repress Bcl2 one, in particular through the activation of p53 [44]. Four conservation laws hold:
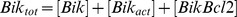


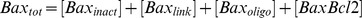


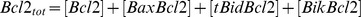


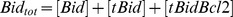



Only seven from the eleven equations of the mathematical model 4–14 need to be solved as the four remaining variables can be computed using those conservation laws.

We subsequently modeled the cell population behavior. Let us denote by 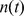 the percentage of surviving cells at time t. No cell division was assumed to occur in presence of staurosporine as the very first effect of most cytotoxic drug consists in stopping the cell cycle [45]. Natural cell death was neglected as almost no apoptosis was observed in either parental or Src-transformed cells in the absence of death stimuli [5]. We considered that apoptosis is irreversibly activated when 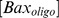 concentration reaches the threshold 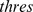 which corresponds to the minimal amount of oligomerized Bax molecules required to trigger the cytochrome C release into the cytosol. This assumption was modeled in equation 15 by a S-shape function which also ensures that the death rate does not grow to infinity. Below is the equation for the percentage of surviving cells:
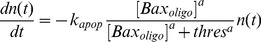
(15)


Parameters 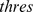, *a* and 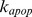 were assumed to be the same for the two cell populations.

At the initial time just before the apoptotic stress, cells were assumed to be in steady state conditions. The initial percentage of surviving cells is 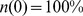. Bik initial concentrations were set to steady-state values computed using equations 1–2. Moreover, we assumed that Bik was entirely under its inactive form so that: 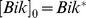, 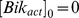, 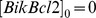. All Bax molecules are assumed to be inactive: 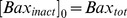, 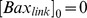 and 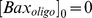. All existing 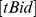 molecules are trapped in complexes with 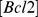: 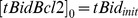 and 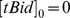. Initial protein concentration of Bid and Bcl2 can be computed using the conservation laws: 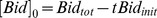 and 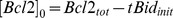. For the sake of simplicity, we considered that no 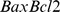 complexes were present at the initial time (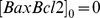) as 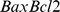 dimers do not play any part in the overall dynamics since we assumed that they do not dissociate.

As previously stated, the considered apoptotic stress consists of an 8-hour-long exposure to staurosporine (2 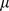
M) which starts at time t = 0. It triggers two molecular events: 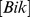 activation into 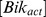 and 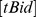 formation representing 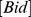 truncation into 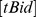. Mathematically, 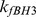 and 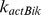 are set to non-zero values at the initial time.

### Estimating parameters of the mitochondrial apoptosis model

Parameters of this model of mitochondrial apoptosis were estimated by fitting experimental data in parental and Src-transformed cells from [5] and integrating biological results from literature. First, we assessed quantitative molar values of considered Bcl-2 family proteins in non-apoptotic conditions as follows. We set Bax total concentration 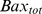 in Src-transformed cells to 100 nM according to [46] in which the authors stated that this was a physiological level in tumor cells. This value is also in agreement with concentration ranges found in the literature [33], [34], [36], [47], [48]. Then, in [46], they found that anti-apoptotic total concentration had to be 6 times higher than that of Bax in order to prevent apoptosis. Therefore, we set 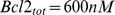 in Src-transformed cells. Concerning Bid total concentration, we assumed 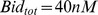 which is in agreement with experimental results from the literature [33], [34], [36], [47]. Finally, tBid initial concentration 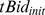 was set to 1 nM since this band was hardly detectable by western-blot (Figure 3). Moreover, this value was consistent with previous modeling results [47].

We then computed protein ratios between parental and Src-transformed cells using immunoblotting data of Figure 3. We experimentally determined that there was a 9-fold higher amount of proteins in the cytosol fraction compared to the mitochondria compartment which allowed us to compute protein ratios of total intracellular quantities (Table 1). As previously stated, Bik protein amount was reduced in Src-transformed cells by a factor of 0.2 compared to parental fibroblasts (Figure 3). This dramatic decrease was the result of the Src-dependent activation of Erk1/2 kinases, leading to Bik phosphorylation, polyubiquitylation and subsequent degradation by the proteasome [5]. Bax steady-state level in non-apoptotic conditions was increased by a factor of 2.1 in Src-transformed cells compared to normal ones and that of Bid was decreased by a factor of 0.77. Concerning antiapoptotic molecules, the sum of Bcl2, Bcl-xL and Mcl-1 quantities was slightly increased in Src-transformed cells by a factor of 1.1 compared to parental ones. Those protein ratios were used to compute molar quantities of considered Bcl-2 family protein total concentrations (Table 1).

Then, we estimated the apoptotic threshold 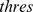 as follows. Quantification of Figure S1d in [5] showed that 38% of BAX molecules at the mitochondria were activated during apoptosis. Previously-described quantification of Figure 3 showed that 33% of BAX total amount were located at the mitochondria, the remaining part being in the cytosol. Therefore, the percentage of activated BAX was set to 33% * 38%/100
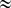
13%. This percentage is in agreement with previous experimental data which suggests that approximately 10–20% of Bax total amount is actually activated during apoptosis [46].

The high intensity of the bands corresponding to Bcl-xL expression in Figure 3 suggested that it might be the predominant antiapoptotic protein in our biological model. Dissociation constant between Bcl-xL and respectively Bik, Bid and Bax was experimentally found to be equal to 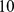 nM, 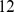 nM and 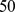 nM [49], [50]. Therefore, we set 
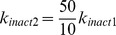
 and 
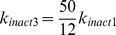
 and only estimated 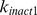. At this point, 10 kinetic parameters still needed to be estimated.

**Figure 6:**
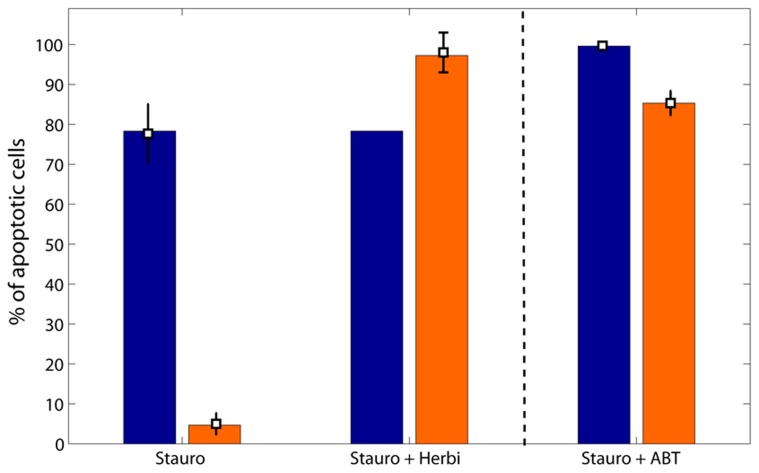
Apoptosis in parental (blue) and Src-transformed (orange) NIH-3T3 cells. Bars represent simulations of the calibrated model of mitochondrial apoptosis. Experimental datapoints for staurosporine given as a single agent or combined with herbimycin were used for parameter estimation. They are reprinted from [5]. Datapoints for the combination of staurosporine with ABT-737 are original data and were used for model validation.

In order to determine those 10 parameters, we fitted experimental data from Figure 6 under constraints inferred from experimental results. We used the three experimental data points of Figure 6 corresponding to exposure to staurosporine as a single agent or combined with herbimycin. We modeled the administration of staurosporine after an exposure to the Src tyrosine kinase inhibitor herbimycin as follows. We assumed that herbimycin was administrated before staurosporine exposure such that the system had time to reach steady state. As previously described, herbimycin exposure was modeled by decreasing 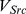 (the maximal velocity of Src-induced Bik ubiquitylation) of 98% of its original value.

Then, we set constraints on state variables as follows. We assumed that 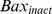 did not decrease below 20% (i.e. the apoptotic threshold 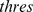) of its initial value within 6 h of staurosporine exposure as approximately 20% of Bax total quantity is activated during apoptosis [9], [46]. Moreover, we ensured that 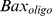 reached the apoptotic threshold in parental cells after 6 to 8 h of exposure to staurosporine as biological experiments showed. Moreover, as Bax oligomerization was assumed to be an autocatalytic process, we expected to obtain 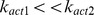. Therefore, in the parameter estimation procedure, we set initial search values for 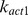 and 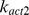 such that 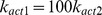. Finally, molecule association rates were searched between 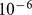 and 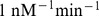 which is a realistic range with respect to the diffusion limit [48]. Estimated parameter values are shown in Table 2.

**Figure 7:**
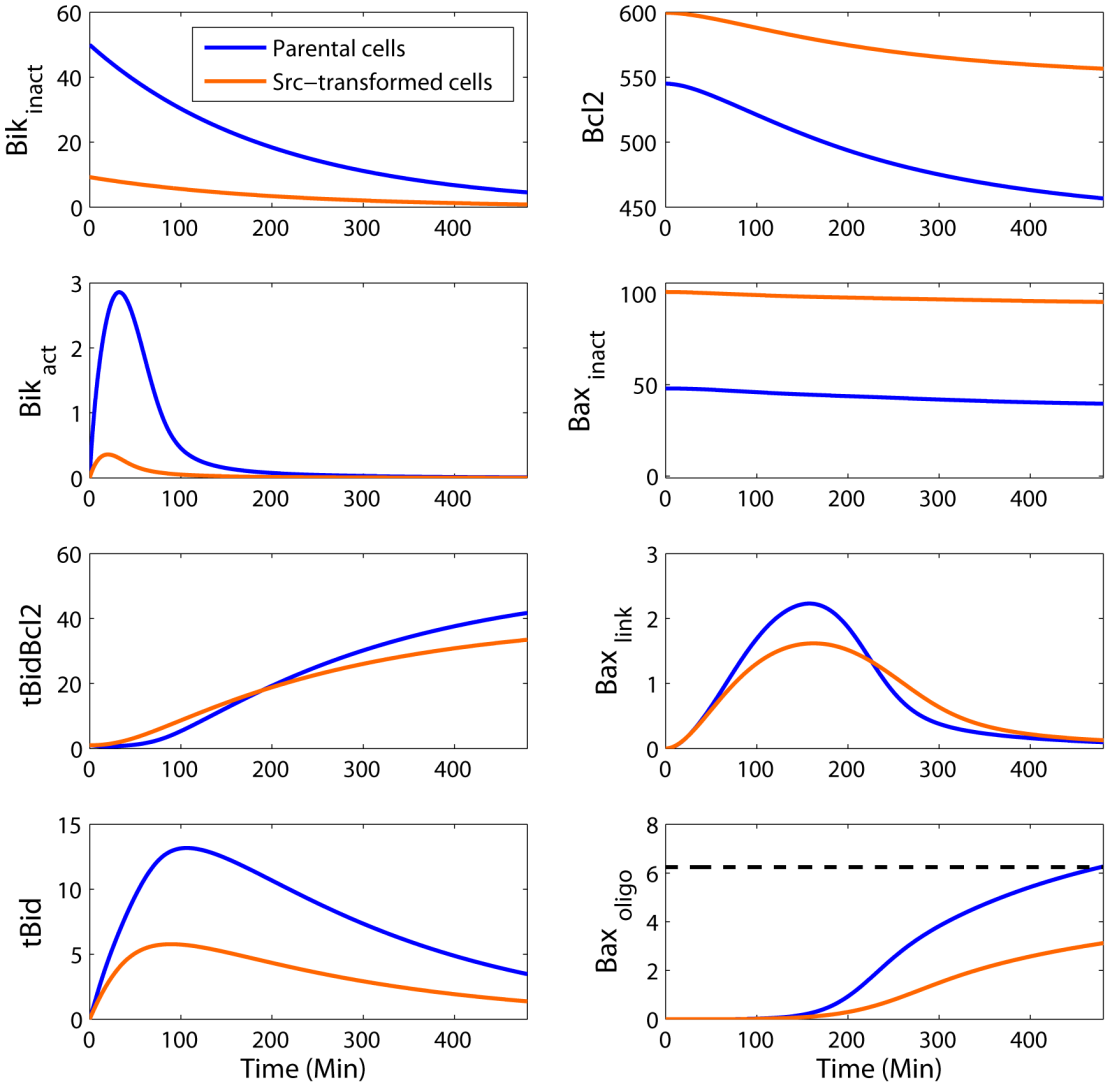
Simulated molecular response to staurosporine exposure of parental (blue) and Src-transformed (orange) NIH-3T3 cells. Higher Bik concentration in normal fibroblasts led to a higher concentration of 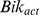 and of free 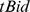. 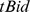 molecules then activated 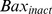 into 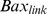 which oligomerized until reaching the apoptotic threshold in control cells but not in Src-transformed cells. Time is expressed in min and concentrations in nM.

The data-fitted mathematical model allowed the investigation of the dynamical molecular response to staurosporine exposure (Figure 7). As expected, higher Bik concentration in normal fibroblasts led to a higher concentration of 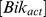 and of free 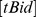 compared to transformed cells. 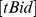 molecules then activated 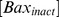 into 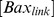 which oligomerized until reaching the apoptotic threshold in parental cells. On the contrary, 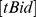 could efficiently be sequestered in complexes with antiapoptotic proteins in Src-transformed cells as a result of the lower level of Bik protein. This perfectly fit the described function of Bik as a sensitizer [51].

Concerning co-administration of staurosporine and herbymicin, the model predicted that this drug combination circumvents the resistance of the cancer cell population in which 99% of cells are apoptotic after 8 hours of exposure to staurosporine (Figure 6). This model behavior was in agreement with experimental data which showed 98% of apoptotic cells in the Src-transformed population. Moreover, the model predicted that an exposure to staurosporine as a single agent or combined with herbimycin lead to the same activity of 80% of apoptotic cells in the parental fibroblasts population.

### Theoretical anticancer therapy optimization and experimental validation

We intended to determine optimal therapeutic strategies for our particular biological system in which parental and Src-transformed NIH-3T3 fibroblasts stand for healthy and cancer cells respectively. In the following, both cell populations are exposed simultaneously to the same drugs, mimicking the *in vivo* situation in which healthy and tumor tissues are *a priori* exposed to the same blood concentrations of chemotherapy agents. From a numerical point of view, identical parameter changes were applied to normal and cancer cells.

First, we investigated the combination of staurosporine with ABT-737, a competitive inhibitor of Bcl-2 and Bcl-xL that were the main antiapoptotic proteins in our cellular model. ABT-737 inhibits free antiapoptotic proteins but also dissociates complexes of anti- and pro-apoptotic proteins. As for herbimycin, we assumed that ABT-737 was administrated before staurosporine such that the system had time to reach steady state. ABT-737 pre-incubation was thus modeled by decreasing Bcl-2 total amount 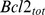 in proportion to ABT-737 concentration and by setting 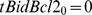 and 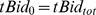 at the initial time.

Interestingly, ABT-737 exposure in the absence of staurosporine (i.e. 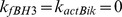) did not result in cell death induction for any dose of ABT-737 in the mathematical model, as experimentally demonstrated [5]. Indeed, in the absence of staurosporine, Bid was not activated into tBid and the low quantity of tBid present in the cells at steady state was not sufficient to trigger Bax oligomerization, even when ABT-737 inhibited all anti-apoptotic proteins. This confirmed that the mathematical model described correctly this cell model that does not behave as a “primed for death model” in which inhibition of anti-death proteins results in death, even in the absence of apoptosis induction. As a reminder, in the primed for death situation, incubation with ABT-737 led to cell death as a consequence of the release of the BH3-only pro-apoptotic proteins that were therefore able to activate Bax. The main difference between the primed for death situation and our model is that apoptosis resistance in the primed for death model primary comes from the overexpression of anti-apoptotic proteins such as Bcl-xL or Bcl2 that are efficiently inhibited by ABT-737 whereas here it comes from the decrease of a pro-death protein in the Src-transformed model.

**Figure 8:**
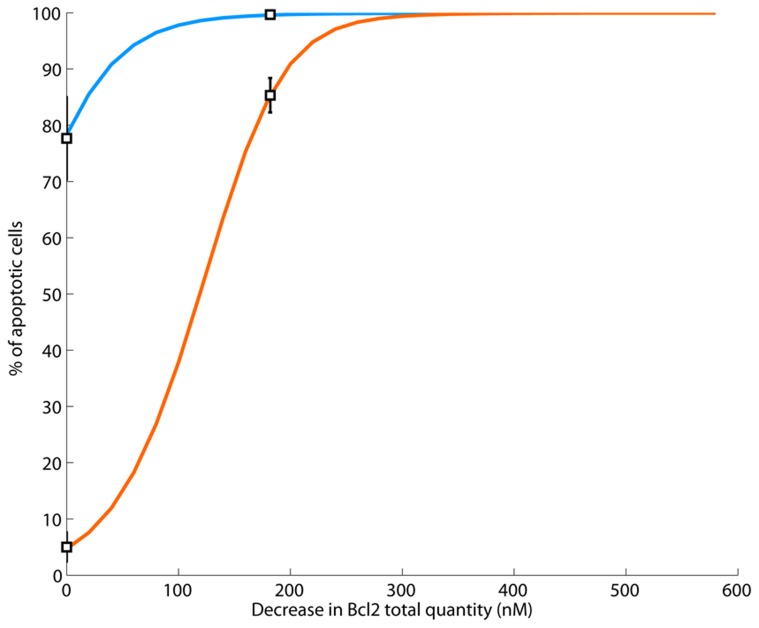
Simulated cell death after staurosporine exposure and preincubation with ABT-737, in parental (blue) and Src-transformed (orange) NIH-3T3 cells. Anti-apoptotic inhibitors such as ABT-737 was modeled as a decrease in the amount of Bcl2 molecules. For any dose of inhibitors, i.e. any decrease of Bcl2 total quantity, simulated apoptosis was greater in parental fibroblasts compared to Src-transformed cells. Moreover, only the administration of high doses managed to circumvent resistance of transformed cells which also induced an extremely high toxicity.

The combination of staurosporine and ABT-737 at any concentration, i.e. for any decrease in Bcl2 total protein amount, was predicted by the model to induce much more apoptosis in parental cells compared to Src-transformed cells and thus to fail in circumventing cancer cells resistance (Figure 8). To experimentally confirm this model prediction, we pre-incubated parental and Src-transformed cells with ABT-737 prior to staurosporine exposure. The resulting death-inducing effect on Src-transformed cells was significantly increased compared to staurosporine alone (Figure 6). However, as anticipated by the model, this drug combination resulted in an extremely high toxicity of 99% of apoptotic cells in the parental fibroblasts population (Figure 6). Those data points were reproduced by the calibrated mathematical model for a predictive decrease of 182 nM in Bcl2 total concentrations in both cell types.

After that, we looked for theoretically optimal therapeutic strategies by applying optimization procedures on the calibrated model of the mitochondrial apoptosis. Optimal strategies were defined as those which maximized efficacy in cancer cells under the toxicity constraint that less than 1% of healthy cells die during drug exposure. We investigated drug combinations that consisted of the exposure to staurosporine after treatment with Src inhibitors, or up- or down-regulators of BCL-2 family proteins. Pre-incubation with inhibitors or activators aimed at modifying the equilibrium of the biological system before exposure to the cytotoxic drug. Src inhibition was simulated by a decrease in 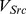 value whereas up- or down-regulation of Bcl-2 family proteins were modeled by modifying the total concentration of the targeted proteins.

**Figure 9:**
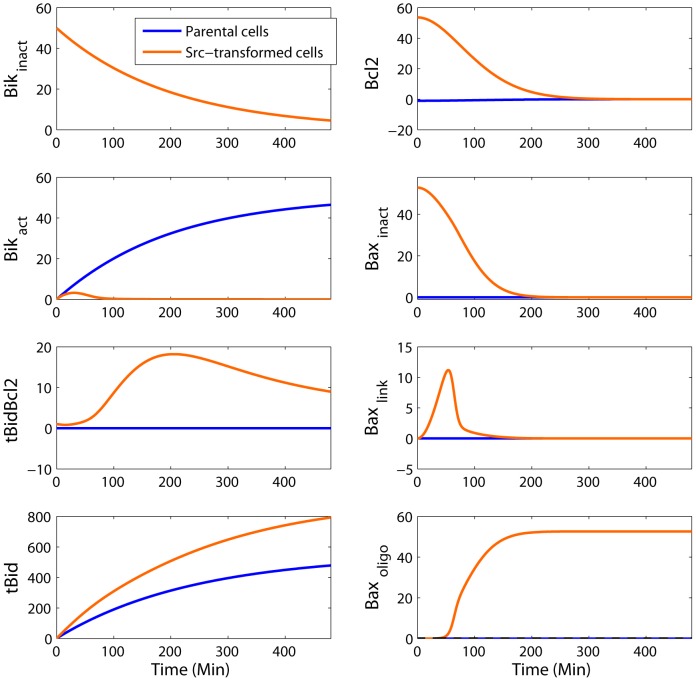
Simulated molecular response to the optimal therapeutic strategy in parental (blue) and Src-transformed (orange) NIH-3T3 cells. The optimal strategy consisted of exposing cells to staurosporine after exposure to four chemicals: an Src inhibitor, which annihilates Bik Src-dependent ubiquitylation in cancer cells, a Bax inhibitor such that Bax quantity is lower than the apoptotic threshold in normal cells, a Bcl2 inhibitor and an agent which enhances Bid transcription. Under this therapeutic strategy, 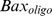 remained under the apoptotic threshold in normal cells and exceeded it in cancer cells. Time is expressed in min and concentrations in nM.

The theoretically-optimal drug combination would consist of administering staurosporine combined with inhibitors of Src, Bax and Bcl2, together with a 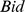 upregulator. The concentration of Bax inhibitor should be set such that Bax total concentration decreases below the apoptotic threshold in healthy cells thus protecting them from apoptosis. As Bax total amount was higher in cancer cells, it would remain high enough to allow these cells to undergo apoptosis. Once healthy cells are sheltered from apoptosis, Bcl2 amount could be decreased, using for instance ABT-737, and 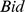 amount increased at the same time without risking any severe toxicity. As expected, the optimal therapeutic strategy also included the suppression of the Src-dependent phosphorylation of Bik in cancer cells, using for instance herbimycin. This drug combination led to 99% of apoptotic cells in the cancer cell population and less than 1% in the parental one where Bax was hardly present (Figure 9, Text S1).

This theoretically optimal strategy involved the administration of a cytotoxic agent combined with four other chemicals, which may not be realistic in the perspective of clinical application. Therefore we hierarchically ranked the considered therapeutic agents by searching for optimal strategies consisting in the combination of staurosporine with only one or two agents. Strategies which satisfied the tolerability constraint (i.e. less than 1% of apoptotic parental cells) and reached an efficacy value of 99% of apoptotic cells all involved Bax downregulation in addition to a second agent among Bcl2 downregulator, 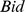 upregulator and Src inhibitor (See Text S1 for more details). Of note, isolated decrease of Bax total amount fulfilled the tolerability constraint but resulted in less than 1% of apoptotic cancer cells.

**Figure 10:**
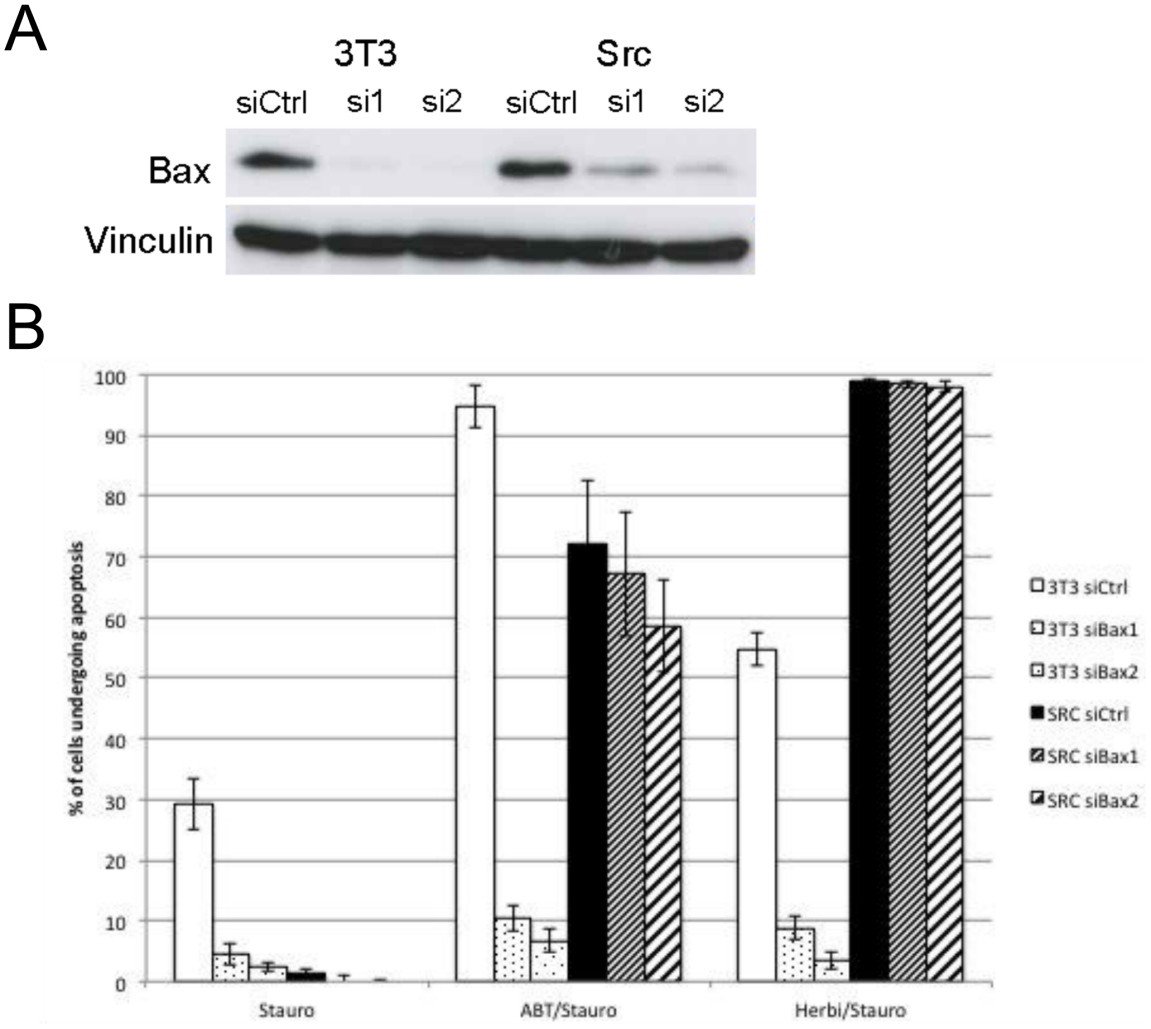
Bax downregulation allowed an advantage to parental cells compared to Src-transformed ones. **A** Bax was downregulated in parental (3T3) and Src-transformed (Src) cells using two different siRNAs (see Material & Methods) such that Bax was not detectable anymore in parental fibroblasts. This was possible since Bax level was higher in transformed cells compared to parental ones (Figure 3) **B** After Bax downregulation, parental cells were highly resistance to exposure to staurosporine given as a single agent, combined with ABT-737 or combined with herbimycin. On the contrary, Src-transformed cells were killed by staurosporine combined with ABT-737 or herbymicin.

Finally, we experimentally validated feasibility of this counterintuitive theoretical strategy. We selected two siRNAs that fully downregulated Bax in parental cells but not in Src-transformed ones (Figure 10 A). Bax knockdown protected parental cells from treatment by staurosporine and ABT737 or staurosporine and herbimycin but not Src-transformed cells (Figure 10 B). Therefore by downregulating Bax in our biological model, we were capable of selectively killing Src-transformed cells.

## Discussion

A combined mathematical and experimental approach was undertaken to study the mitochondrial pathway of apoptosis in parental and Src-transformed NIH-3T3 cells. First, a mathematical model for Bik kinetics in normal and apoptotic conditions was built. It took into account Bik ubiquitylation and further proteasomal degradation that Src-dependent Bik phosphorylation stimulated in Src-transformed cells. Then, we designed a mathematical model of the mitochondrial pathway of apoptosis which only involved the proteins that participated in apoptosis induction in the studied biological model. Interestingly, this mathematical model was quite simple, with only one effector, Bax, two BH3-only proteins, Bik (a sensitizer) and tBid (a direct Bax activator), and a pool of antiapoptotic proteins which were all described as behaving identically toward Bax, Bik and tBid [38].

Several published works propose mathematical modeling of apoptosis. Some of them model all pathways to apoptosis from the death stimulus to the actual cell death [52]–[54], other focus on the caspase cascade leading to apoptosis [55]. Molecular modeling of the mitochondrial pathway was achieved in several works [47], [48], [56]–[59]. Those models being conceived to address other biological issues, we had to build a new mathematical model that was tailored to our particular problematic and aimed at optimizing anticancer therapies in the specific case of Src transformation.

Exploring Bik kinetics upon apoptosis induction led to the interesting prediction that the inhibition of Bik degradation might not allow its accumulation above a threshold that would induce apoptosis in the experimentally-demonstrated time range. This was validated by immunoblotting that established that Bik concentration was not changed upon apoptosis induction by staurosporine. Therefore, we looked for another explanation that might support these observations. A possibility was that Bik might undergo activation upon apoptosis induction. Activation of BH3-only proteins has already been described and can depend on phosphorylation/dephosphorylation as observed for Bad or Bim or on proteolytic activation as for Bid. These post-translational modifications usually result in a change of cell compartment, from cytosol to mitochondria for Bad, from cytoskeleton to mitochondria for Bim. Therefore, we looked for a clue pointing towards Bik activation during apoptosis induction. Indeed, we observed that a significant part of Bik translocated from the ER, which is its normal location, to mitochondria upon staurosporine treatment. This could be due to the release of Bik from a protein complex with the ER protein GRP78 as described in [41]. However, we could not rule out that this relocation might also be linked to the hyperfusion and subsequent mitochondria fission process observed upon a number of apoptosis stresses [60] or more specifically associated with Bik [61].

The mathematical model of apoptosis induction was used to explore anticancer therapies. First, it confirmed that Src inhibitor circumvented resistance to staurosporine exposure of Src-transformed cells, which was experimentally demonstrated. It also showed that this therapeutic strategy did not give any specific advantage to parental cells. It also predicted that inhibitors of antiapoptotic proteins should not be co-administered with staurosporine in our particular biological system as a result of the slightly lower antiapoptotic protein concentration in Src-transformed cells compared to parental ones. This model prediction was experimentally validated. Indeed, here, resistance to apoptosis comes from the decrease in a pro-apoptotic protein in Src-transformed cells and not from the overexpression of antiapoptotic proteins as frequently observed, which explains why a drug inhibiting antiapoptotic proteins could not target specifically transformed cells.

We then investigated theoretically-optimal therapeutic strategies. Interestingly, all optimal drug combinations took advantage of the observed Bax overexpression in Src-transformed cells. The optimal therapeutic strategies consisted of the combination of a cytotoxic agent for the induction of apoptosis (staurosporine in the model) with Bax downregulator and one agent among Src inhibitor, Bcl2 inhibitor or tBid upregulator. We experimentally validated this counterintuitive prediction by demonstrating in our biological model that Bax knockdown could protect parental cells but not Src-transformed cells from combinations of staurosporine with antiapoptotic protein or Src tyrosine kinase inhibitor.

This strategy might be challenging in clinics since siRNA targeting Bax would need to have the exact same pharmacokinetic properties as inhibitors of tyrosine kinase or of antiapoptotic proteins to target all the places where there is expected to be cytotoxicity. Moreover it would be of importance to also downregulate Bak, due to redundancy between Bax and Bak [62]. It would also be of interest to check for Bax/Bak upregulation in various tumors. Therefore, this study supports the need for further development of Bax/Bak inhibitors that might open a therapeutic window for kinase or antiapoptotic protein inhibitors that would otherwise be globally harmful for the patient when combined with a cytotoxic treatment.

## Materials and Methods

### Cell culture and drug exposure

Parental and v-src transformed NIH-3T3 cells were cultured as described in [5]. When indicated, cells were exposed to staurosporine (2 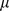
M) during 8 hours and pre-incubated overnight with herbimycin (1 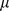
M) or ABT-737 (1 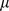
M) prior to staurosporine exposure.

### Immunoblotting

Immunoblotting was performed as described in [5].

### Confocal microscopy

For the analysis of Bik relocalization to mitochondria upon staurosporine exposure, cells were pre-incubated with Mitotracker-red (1∶10,000) for 20 min before a 20 min washing in fresh medium. Then, cells were fixed by addition of paraformaldehyde (4%). Mitotracker-red background was removed by a 10 min incubation in acetone at −20°C prior further processing according to standard procedures. Endogenous Bik was detected by the specific anti-Bik-BH3 antibody (1∶200). The secondary antibody (Molecular Probe) was labeled with FITC. Nuclei were stained with Hoechst-33342 dye (1∶50,000) in the mounting medium. Images were acquired by confocal microscopy on a Axiovert 100 M, LSM510 (Zeiss) using a Plan Apochromat 63x/1.4 Oil DIC objective. Colocalization of Bik fluorescence and mitotracker staining was quantified using ImageJ software in 30 single cells treated with staurosporine for 2 h.

### Bax downregulation

siBax1 (MMC.RNAI.N007527.12.2) and siBax2 (MMC.RNAI.N007527.12.4) were purchased from Integrated DNA Technologies. 100.000 cells were transfected with 10 nM siRNA using Lipofectamine RNAimax (Life Technologies). 48 h later, cells were treated overnight either by ABT737 or herbimycin. The day after, apoptosis was induced by staurosporine treatment (exposure to 2 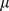
M during 8 hours).

### Mathematical modeling and parameter estimation

Matlab *ode15s* solver was used to solve the systems of ordinary differential equations. Parameter estimation for Bik kinetics model consisted of a least-square approach in which minimization tasks were performed by the CMAES algorithm ([63], Text S1). Parameter estimation for the mitochondrial apoptosis model involved the design of a cost function which was also minimized by the CMAES algorithm. This cost function was the sum of a Least square term accounting for the fitting of experimental data of Figure 6 and a term for the constraints described in the Results section. This term was equal to zero when all constraints were satisfied and to a thousand times the number of unsatisfied constraints otherwise [64].

### Therapeutics optimization

Optimization procedures for the design of therapeutic strategies consisted of maximizing efficacy under the constraint of toxicity not exceeding a tolerability threshold. In order to address this issue we minimized a cost function which was the sum of two terms. The first one consisted of the percentage of surviving cells in the cancer cell population which has to be minimized. The second term was equal to zero when the toxicity constraint was satisfied and took a high value (e.g. 1000) otherwise. Minimization tasks were performed by the CMAES algorithm [63].

## Supporting Information

Text S1 This text contains details about parameter estimation of the Bik kinetics model as well as additional information on numerical simulations for therapeutics optimization.
(PDF)
