# Supplementary material for: Data-Driven Modeling of Src Control on the Mitochondrial Pathway of Apoptosis: Implication for Anticancer Therapy Optimization
Source: PLoS Comput Biol. 2013 Apr 4;9(4):e1003011. doi: 10.1371/journal.pcbi.1003011 (PMC3616992; doi:10.1371/journal.pcbi.1003011)
Supplement: Text S1 — This text contains details about parameter estimation of the Bik kinetics model as well as additional information on numerical simulations for therapeutics optimization. (PDF) [file pcbi.1003011.s001.pdf]

Supporting information S1

1 Parameter estimation for Bik kinetics

We estimated parameters of Bik degradation as follows. First, we assumed that Bik spontaneous ubiquitylation occurred at the same rate in control and transformed fibroblasts and therefore looked for a unique k_{ubBIK} parameter value. Parameters of Src-dependent Bik degradation were denoted V_{Src}^{normal} and K_{Src}^{normal} in parental cells and V_{Src} and K_{Src} in transformed cells. Parameter estimation was performed by fitting data of Figure 2B. Those data points are expressed as percentages of Bik initial concentration $Bik^{initial}$ which is unknow and has to be estimated as an additional parameter.

In these experiments, Bik synthesis was completely inhibited by the mean of two drugs: cells were incubated both with actinomycin D which is an inhibitor of mRNA synthesis and cycloheximide which inhibits proteic synthesis. Therefore we assumed that protein formation was completely inhibited for the whole duration of the experiments, i.e. $k_{fBIK} = 0$. Bik equation then becomes:

$$\frac{d[Bik]}{dt} = -k_{ubBik}[Bik] - \frac{V_{Src}[Bik]}{K_{Src} + [Bik]}$$

where Bik concentration is expressed in nM. At the initial time, Bik was set to $Bik^{initial}$ in parental and Src-transformed cells. We computed the best-fit parameter values for both cell types by a least square approach using the CMAES algorithm for the minimization of the cost function. This gave:

$$\begin{aligned} Bik^{initial} &= 5.9nM \\ k_{ubBIK} &= 0.0036min^{-1} \\ V_{Src}^{normal} &= 0min^{-1}.nM^{-1} \\ V_{Src} &= 10.8min^{-1}.nM^{-1} \\ K_{Src} &= 671nM \end{aligned}$$

2 Therapeutics optimization

We determined theoretically optimal therapeutic strategies by applying optimization procedures on the data-fitted model of the mitochondrial pathway of apoptosis. We investigated drug combinations which consisted in an exposure to staurosporine after pre-incubation with Src inhibitors, or with up- or down-regulators of Bcl2 family protein amounts.

The optimization procedure consisted in searching the optimal values of four parameters corresponding to the pharmacological activity on Bax, Bcl2, Bid and Src. For Bax, Bcl2 and Bid, factors were added to their respective total amount. For Src, the parameter V_{Src} which is the maximal velocity of Src-dependent Bik ubiquitylation was multiplied by the indicated factor. Bax factor was searched in [-Bax_{tot}, 500], Bcl2 factor in [-$Bcl2_{tot}$, 500], Bid factor in [-Bid_{tot_cancer}, 500] and Src factor in [0.0001 500].

The theoretically-optimal drug combination consisted in administering staurosporine combined to inhibitors of Src, Bax and Bcl2, together with a *Bid* upregulator. Bax factor was equal to -Bax_{tot} which led to Bax concentration in parental cells equal to zero thus protecting them from apoptosis. As Bax total amount was higher in cancer cells, it remained high enough to allow these cells to undergo apoptosis. Once healthy cells were sheltered from apoptosis, Bcl2 amount could be decreased of -$Bcl2_{tot}$ and *Bid* amount increased of 500 nM (i.e. the maximal allowed value) without risking any severe toxicity. As expected, the optimal therapeutic strategy also included the suppression of the Src-dependent phosphorylation of Bik as Src factor was equal to 0.0001. This drug combination led to 99% of apoptotic cells in the cancer cell population and less than 1% in the parental one where Bax was hardly present (Table S1).

This theoretically optimal strategy involved the administration of a cytotoxic agent combined with four other chemicals, which may not be realistic in the perspective of clinical application. Therefore we hierarchically ranked the considered therapeutic agents by searching for optimal strategies consisting in the combination of staurosporine with only one or two agents. We computed efficacy on Src-transformed cells and toxicity on parental cells for each possible combination in which drugs were given at the same dose as in the previously-determined optimal combination (Table S1).

Strategies which satisfied the tolerability constraint (i.e. less than 1% of apoptotic parental cells) and reached an efficacy value of 99 % of apoptotic cells all involved Bax downregulation in addition to a second agent among Bcl2 downregulator, *Bid* upregulator and Src inhibitor (Table S1). Isolated decrease of Bax total quantity fulfilled the tolerability constraint but resulted in less than 1% of apoptotic cancer cells.

Table S1: **Simulated toxicity and efficacy of staurosporine exposure after incubation with indicated up- and downregulators.**

Bax factor (nM)	Bcl2 factor (nM)	Bid factor (nM)	Src factor	**Parental cells (% of apoptotic cells)**	**Src-transformed cells (% of apoptotic cells)**
1 agent					
$-Bax_{tot}$	0	0	1	0	0.005
0	$-Bcl2_{tot}$	0	1	99.99	99.98
0	0	500	1	99.13	28.9
0	0	0	0.0001	76.97	75.32
2 agents					
$-Bax_{tot}$	$-Bcl2_{tot}$	0	1	0	99.99
$-Bax_{tot}$	0	500	1	0	27.66
$-Bax_{tot}$	0	0	0.0001	0	73.05
0	$-Bcl2_{tot}$	500	1	99.99	99.99
0	$-Bcl2_{tot}$	0	0.0001	99.99	99.99
0	0	500	0.0001	99.1	99.03
4 agents					
$-Bax_{tot}$	$-Bcl2_{tot}$	500	0.0001	0	99.9

We investigated drug combinations which consisted in an exposure to staurosporine after pre-incubation with Src inhibitors, or with up- or down-regulators of Bcl2 family protein amounts. For Bax, Bcl2 and Bid, factors where added to their respective total amount. For Src, the parameter V_{Src} which is the maximal velocity of Src-dependent Bik ubiquitylation was multiplied by the indicated factor.

3 Steady State study

The mathematical model of mitochondrial apoptosis admits three kinds of steady states (Table S2). Steady state 1 corresponds to a complete inhibition of BIK_{mito} and $BH3_a$ by BCL2. Moreover, a part of BAX proteins has not been activated. In steady state 2 and 3, BCL2 is completely consumed into complexes with BIK_{mito}, $BH3_a$ and BAX_{link}. BAX_{inact} has been entirely activated into BAX_{oligo}. In steady state 2, some $BH3_aBCL2$ complexes still remain in the cytosol whereas BIK_{mito} proteins have been entirely consumed. In steady state 3, this is the opposite situation. Steady states 2 and 3 do not seem realistic since all BAX molecules are activated whereas only 10 to 20% of BAX total amount are effectively activated during apoptosis [9,46].

Table S2: **Steady states of the model of the mitochondrial pathway to apoptosis.**

	**Steady state 1**	**Steady state 2**	**Steady state 3**
$[BIK]^*$	0	0	0
$[BIK_{mito}]^*$	0	0	arbitrarily set
$[BIKBCL2]^*$	BIK_{tot}	BIK_{tot}	$BIK_{tot} - [BIK_{mito}]^*$
$[BID]^*$	0	0	0
$[BH3_a]^*$	0	arbitrarily set	BID_{tot}
$[BH3_aBCL2]^*$	BID_{tot}	$BID_{tot} - [BH3_a]^*$	0
$[BAX_{inact}]^*$	arbitrarily set	0	0
$[BAX_{link}]^*$	0	0	0
$[BAX_{oligo}]^*$	arbitrarily set	$BAX_{tot} - [BAXBCL2]^*$	$BAX_{tot} - [BAXBCL2]^*$
$[BAXBCL2]^*$	$BAX_{tot} - [BAX_{inact}]^* - [BAXBCL2]^*$	$BCL2_{tot} - [BIKBCL2]^* - [BH3_aBCL2]^* - [BAXBCL2]^*$	$BCL2_{tot} - [BIKBCL2]^* - [BH3_aBCL2]^* - [BAXBCL2]^*$
$[BCL2]^*$	$BCL2_{tot} - [BIKBCL2]^* - [BH3_aBCL2]^* - [BAXBCL2]^*$	0	0
